# Impact of fiber molecular structure on resistance to digestion using the infogest and rat small intestine extract protocols

**DOI:** 10.1007/s00394-025-03853-0

**Published:** 2025-12-06

**Authors:** Fatma Boukid, Pablo Méndez-Albiñana, Alejandro Sánchez-Baca, Mar Villamiel

**Affiliations:** 1 ClonBio Group LTD, 6 Fitzwilliam Pl, Dublin, Dublin, D02 XE61 Ireland; 2 Department of Physiology, School of Medicine, Universidad Autónoma de Madrid, Madrid, Spain; 3 Group of Chemistry and Functionality of Carbohydrates and Derivatives, Food Science Research Institute (CIAL) (CSIC-UAM), Madrid, Spain

**Keywords:** Dietary fibers, Polysaccharide hydrolysis, Molecular weight, In vitro digestion, Simple sugar release, Carbohydrate profile

## Abstract

**Purpose:**

Dietary fibers differ in their molecular structure, which influences their breakdown under digestive conditions. This study investigates how fiber molecular structure affects resistance to digestion using in vitro models.

**Methods:**

High-, medium-, and low-molecular-weight (Mw) corn arabinoxylans, resistant maltodextrin, and inulin were characterized by carbohydrate composition following acid hydrolysis and analyzed for sugar release during simulated digestion using the standardized InfoGest protocol and rat small intestinal extract (RSIE).

**Results:**

High-Mw corn arabinoxylan (208.8 kDa) remained largely stable, with minor, non-significant increases in glucose and galactose. Medium- (25.5 kDa) and low-Mw (2.5 kDa) arabinoxylans showed partial hydrolysis, with increases in trisaccharides and maltose and decreases in glucose and arabinose. Resistant maltodextrin (1.9 kDa) displayed significant increases in glucose, trisaccharides, and maltose. Inulin (0.75 kDa) remained mostly intact, with only partial decreases in fructose and sucrose. Sugar release during 4 h of simulated digestion followed these trends: high-Mw arabinoxylan released 11.1 mg/g, medium-Mw released 5.20 mg/g/h, low-Mw released 9.84 mg/g, resistant maltodextrin released 6.83 mg/g/h, and inulin released 59.9 mg/g.

**Conclusion:**

These findings demonstrate that fiber structural variations critically influence the degree of hydrolysis during digestion and the resulting simple sugar release.

**Supplementary Information:**

The online version contains supplementary material available at 10.1007/s00394-025-03853-0.

## Introduction

Dietary fibers are complex plant-based carbohydrates that are resistant to digestion by human enzymes in the small intestine [53], and pass largely intact into the colon, where they can influence gut microbiota and fermentation [27]. Fiber intake supports gastrointestinal, cardiovascular, and metabolic health [2, 39]. The physiological effects of dietary fibers depend on their structural properties, including molecular weight (Mw), branching, and solubility. These features determine how resistant the fibers are to enzymatic digestion and shape their overall functional benefits [13, 29, 50].

Dietary fibers exist in diverse forms, originating from natural sources such as fruits, vegetables, grains, and legumes, as well as from processed or modified sources [35]. These fibers differ in key structural characteristics such as Mw and branching, which determine their physical behavior and digestibility [17, 61]. In addition, some fibers undergo chemical modifications, including acetylation, methylation, or enzymatic cross-linking, which can further alter their solubility, stability, and susceptibility to enzymatic degradation [31, 62]. For instance, cellulose, hemicellulose, and pectin are naturally occurring fibers found in plant cell walls, whereas inulin, resistant starch, and resistant maltodextrin are processed fibers developed for specific functional purposes [1, 7, 55]. These structural variations govern how fibers resist enzymatic breakdown and modulate the rate and extent of sugar release during digestion, which is central to understanding their physiological and metabolic effects [38, 44].

Among these fibers, arabinoxylans have recently emerged as an important category, particularly those derived from wheat and corn [40, 73]. Arabinoxylans are hemicellulosic polysaccharides that constitute a major fraction of non-starch polysaccharides in cereal grains [6]. They are increasingly recognized for their potential health benefits and are becoming more relevant in the food industry [49]. This growing interest is largely due to due to their availability as by-products of the milling, ethanol, and oil industries, and resulting in transforming agricultural residues into value-added ingredients [69]. Thus, the increasing use of arabinoxylans represents both a nutritional and sustainability opportunity, contributing to dietary fiber enrichment while reducing industrial waste [36, 70, 71].

Resistance to digestion is a key determinant of fiber functionality [9, 45]. Fibers that resist enzymatic digestion in the small intestine pass into the colon, where microbial fermentation produces short-chain fatty acids (SCFAs) that confer additional health benefits contributing to well-documented metabolic benefits [16, 34]. Conversely, fibers with more accessible structures, such as those with lower Mw or higher solubility, may be partially or fully digested earlier in the gastrointestinal tract [21]. Understanding how fiber structure influences digestion is critical for optimizing their functional and metabolic effects, including impacts on the gut microbiome, blood sugar regulation, and lipid metabolism [27, 43, 68].

Comparative studies systematically linking fiber molecular structure to digestive resistance remain scarce. Specifically, the influence of structural features such as Mw, branching, and solubility on enzymatic resistance has not been systematically assessed across multiple fiber types, including inulin, resistant starch, resistant maltodextrin, and arabinoxylans [11, 25,28]. Moreover, many studies rely on simplified in vitro models lacking standardized digestion protocols or detailed acid hydrolysis steps to quantify monosaccharide release. Thus, the focus of the most common approach to simulate the small intestinal digestion has been dedicated to proteins, lipids and starch, by using pancreatic enzymes from porcine origin, salivary enzymes and microbial enzymes, which could not reflex most of the carbohydrase activities of the whole small intestine. These enzymes cannot completely hydrolyze digestible saccharides since they do not represent the fully complex enzymatic environment of the small intestine, mainly because of the absence of the brush border enzymes of the enterocytes. As a result, digestible saccharides that are not fully degraded are considered as non-digestible carbohydrates, which lead to an inaccurate determination of the digestion resistance of these carbohydrates. A promising in vitro digestibility method of dietary carbohydrates using rat small intestinal extract (RSIE) has questioned the belief that soluble fibers reach the distal portions of colon without alterations [22]. The degree of digestion of inulin-type fructans and fructooligosaccharides has been studied using RSIE demonstrating the high resistance of fructosyl-fructose bonds [23]. Starch, dextran, pectin and modified citrus pectin were subjected to intestinal digestion following InfoGest protocol and a rat small intestine extract (RSIE) treatment. The results demonstrated that InfoGest method underestimates the significance of carbohydrates hydrolysis at the small intestine, thus indicating that RSIE is a very reliable and useful method for a more realistic study of polysaccharides digestion [25].

This study addresses these gaps by applying the standardized InfoGest 2.0 in vitro digestion model in combination with RSIE to investigate how structural variations among fibers influence their breakdown during digestion. Importantly, this study is among the first to evaluate arabinoxylans, resistant maltodextrin, and inulin using a combined Infogest + RSIE approach. While the combination of InfoGest and RSIE has been applied to other polysaccharides, such as starch and pectins [23, 25]), , its use with these fiber types provides a more physiologically relevant assessment of carbohydrate digestion, including oligo- and polysaccharides. We hypothesize that fibers with higher molecular weight and more complex branching will exhibit greater resistance to enzymatic digestion and slower sugar release. By systematically comparing multiple fibers under standardized digestion conditions, this approach also provides additional insights into glucose and other sugar release profiles. The expected outcomes include a detailed mechanistic understanding of how molecular structure influences digestive behavior, which can guide the design of functional foods enriched with structurally optimized fibers.

## Materials and methods

### Fiber samples

Table 1 presents the characterization of various dietary fiber samples used in this study. The fibers were sourced from different providers, each with distinct molecular weight ranges and properties, which may influence their digestive stability and functionality. All fibers were analyzed in duplicate.

**Table 1:** General characteristics of the different arabinoxylans, resistant maltodextrin, and inulin samples

Feature	High molecular weight corn arabinoxylan	Medium molecular weight corn arabinoxylan	Low molecular weight corn arabinoxylan	Resistant Maltodextrin	Inulin
Manufacturer	Pannonia Bio Zrt (Hungary)	Pannonia Bio Zrt (Hungary)	Pannonia Bio Zrt (Hungary)	Tate & Lyle (United Kingdom)	Naturemind (China)
Moisture content (%)	0.05	0.11	0.07	0.06	0.02
pH (1% w/v)	9.91 ± 0.001	6.21 ± 0.01	10.59 ± 0.01	5.28 ± 0.01	5.73 ± 0.001
Protein content (%)	2.12	0.74	0.67	0.0	0.0

### Determination of molecular weight

Estimation of relative molecular weight (Mw) of the studied fibers was conducted by Size Exclusion Chromatography (SEC) according to Muñoz-Almagro et al. [48], using an Agilent Technologies 1220 Infinity LC System (Agilent Technologies, Germany), equipped with TSK-GEL columns (G5000 PWXL, 7.8 × 300 mm, particle size 10 μm; G2500 PWXL, 7.8 × 300 mm, particle size 6 μm) (Tosoh Bioscience, Stuttgart, Germany). Firstly, samples were diluted in Milli-Q water. After that, 20 µL were eluted with 0.01 M ammonium acetate at a flow rate of 0.5 mL/min for 50 min at 30 °C, using an ELSD 1260 Infinity (B¨ oblingen, Germany) at 30 °C for detection. For calibration and quantification purposes, a set of pullulan standards was used, specifically pullulans with Mw values of 805, 200, 10, 1.3, and 0.34 kDa [48]. Standard curves of pullulans for Mw estimation were obtained considering the logarithm of Mw versus the corresponding elution volume. The samples were analyzed in duplicate.

### Analysis of carbohydrates

Two types of carbohydrate analysis were performed: determination of the soluble fraction and monomeric composition, the later after acid hydrolysis. For the soluble fraction, 30 mg of sample were added to 1 mL of Milli-Q water, stirred for 30 min at room temperature, and centrifuged at 10,000 × g for 3 min. From the supernatant, 0.25 mL were mixed with 400 µL of phenyl-β-D-glucoside (internal standard, 0.5 mg/mL) and evaporated to dryness prior to derivatization. For monomeric composition, 30 mg of sample were hydrolyzed with 1.5 mL of 2 M trifluoroacetic acid (TFA) at 110 °C for 4 h. This condition was chosen because 2 M TFA at 110 °C for 4 h is a standard hydrolysis protocol that efficiently depolymerizes complex polysaccharides into their constituent monosaccharides while minimizing sugar degradation [18, 65]. After hydrolysis, the acid was removed by evaporation, and 400 µL of phenyl-β-D-glucoside (0.5 mg/mL) was added, followed by drying prior to derivatization. Derivatization to trimethylsilylated oximes (TMSOs) was performed following Berüter [4] with slight modifications: samples were mixed with 400 µL of internal standard, dried, reacted with 300 µL hydroxylamine chloride in pyridine (2.5%, w/v) at 70 °C for 30 min to form oximes, then silylated with 300 µL hexamethyldisilazane (HMDS) and 30 µL TFA at 50 °C for 30 min with agitation. Derivatized samples were centrifuged at 10,000 × g for 2 min, and supernatants were injected into GC-FID. Analyses were performed on Agilent 7820–7890 A gas chromatograph equipped with FID and autosamplers, using VF-5HT or DB-5HT columns (30 m × 0.25 mm × 0.10 μm, 5% phenyl methylpolysiloxane) with nitrogen as the carrier gas at 1 mL/min. Oven programs ranged from 120 to 150 °C to 300–380 °C at 3 °C/min, depending on sample type, with injector and detector temperatures between 280 and 385 °C and split injection mode (1:5–1:20). Data processing was performed using Agilent ChemStation software, and quantitative analysis used the internal standard method with β-phenyl-glucoside. Calibration standards included xylose, arabinose, rhamnose, galactose, mannose, glucose, galacturonic acid, sucrose, fructose, maltose, and maltotriose (0.005–2 mg/mL). All analyses were conducted in duplicate.

Acid hydrolysis was used to determine the monomeric composition of the fibers, providing a baseline structural characterization. In vitro digestion was performed to evaluate the fibers’ resistance to enzymatic breakdown under simulated gastrointestinal conditions. These analyses were treated separately, allowing us to understand how fiber structural features relate to functional digestibility without implying direct cross-validation.

### In vitro digestion of fibers

All enzymes and reagents used in the in vitro digestion, including porcine pepsin, porcine pancreatin, bile salts, and CaCl_2_, were purchased from Sigma-Aldrich (St. Louis, MO, USA) and used without further purification (Table S1). The digestion process of fibers was performed following a previously published method described [25], using a static in vitro digestion model based on the InfoGest 2.0 protocol [8]. Briefly, 0.3 g of each fiber (arabinoxylans, resistant maltodextrin and inulin) were dissolved in 10 mL of distilled water to prepare solutions at 30 mg/mL (3%, w/w). These solutions were then subjected to the gastric and intestinal digestion phases. For the gastric phase, these solutions were added with an equal volume of simulated gastric fluid (SGF). Porcine pepsin was added to achieve a final concentration of 2000 U/mL, followed by the addition of 5 µL of 0.3 M CaCl₂. The pH was adjusted to 3.0, and the mixture was incubated at 37 °C in an orbital thermomixer with continuous agitation (750 rpm) for 2 h. The initial sampling time (0 h) was defined as the exact moment when each compound was mixed with the simulated gastric fluid (SGF, pH 3.0), corresponding to the onset of the gastric phase, i.e., the moment equivalent to food entry into the stomach. This definition was adopted to ensure clarity in the interpretation of digestion time points throughout the sequential in vitro digestion process (2 h gastric + 2 h intestinal (1 h intestinal + 1 h RSIE)). For the intestinal phase, the gastric digesta of each carbohydrate was mixed with an equal volume of simulated intestinal fluid (SIF). Porcine pancreatin and bile salt were added to reach final concentrations of 100 U/mL (trypsin activity) and 10 mM, respectively. Additionally, 40 µL of 0.3 M CaCl₂ were added, and the pH was adjusted to 7.0. Digestions were conducted in an orbital thermomixer at 37 °C with continuous agitation for 1 h. Following this, carbohydrate samples underwent further digestion with rat small intestine extract (RSIE). Each carbohydrate was prepared at a final concentration of 0.5 mg/mL and mixed with RSIE to achieve a final RSIE concentration of 40 mg/mL. The reactions were carried out in an orbital thermomixer at 37 °C and 750 rpm for 1 h. Digestion was halted by heating the samples (95 °C for 10 min). All samples were analyzed in biological triplicate, each injected in technical duplicate.

Once fibers were digested, soluble carbohydrates were analyzed before and after the digestion following the GC-FID determination indicated in Sect. Analysis of carbohydrates. Background carbohydrates from the enzyme preparations (e.g., lactose) were quantified and subtracted from all sample measurements across the gastric, intestinal, and RSIE digestion phases (Table S2).

### Rate of simple sugar release after digestion

The release of simple sugars (i.e., glucose, galactose, fructose, and mannose), assessed by GC-FID, was quantified by the amount of sugar released during the digestion process. The rate of sugar release was normalized to sample weight and calculated as:

$$\begin{aligned} & Rate~of~simple~sugar~release~\left( {{\mathrm{mg}}/{\mathrm{g}}/{\mathrm{hour}}} \right) \\ & = \frac{{Simple~sugars~at~4h - ~Simple~sugars~at~0h}}{{4h}} \\ \end{aligned} $$

Simple sugar values at 0 h and 4 h reflect the free sugars at the start and after 4 h of digestion. Because all values are expressed relative to sample mass (mg/g), the rate calculation is already normalized.

### Statistical analysis

The statistical analysis was conducted using the Statistical Package for Social Sciences software (IBM SPSS Statistics, Version 25.0, IBM Corp., Chicago, IL, USA). All data are presented as mean ± standard deviation (SD). All samples were analyzed in biological triplicate (*n* = 3), with each injected in technical duplicate for analytical precision. Statistical significance between groups was determined using a one-way analysis of variance (ANOVA), followed by Duncan’s post hoc test for pairwise comparisons. The statistical outcomes are interpreted primarily as indicative of strong physicochemical trends observed between the characterized structural groups. A p-value of less than 0.05 was considered statistically significant.

## Results and discussion

### Fiber molecular weight

**Fig. 1:**
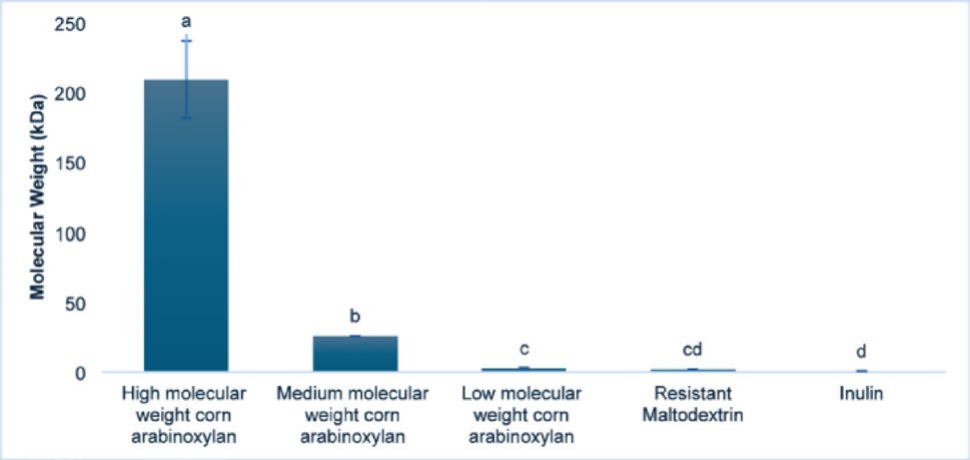
Molecular weight (Mw) of the different arabinoxylans, resistant maltodextrin and inulin samples estimated by HPSEC-ELSD

As illustrated in Fig. 1, the Mw analysis of the fiber samples demonstrated significant differences among the polysaccharides. The full chromatographic profiles obtained by HPSEC-ELSD are provided in Figures S1, S2, S3, S4. High Mw corn arabinoxylan had the greatest molecular size at 208.8 kDa. This value is consistent with some reported values for native corn bran arabinoxylan, which can be rather high. For example, previous research reported Mw values of 200–300 kDa for native arabinoxylan extracted from corn bran [32, 72]. In contrast, medium Mw corn arabinoxylan showed a significantly lower estimation (25.5 kDa), aligning with literature values for hydrolyzed arabinoxylan fractions, usually found between 10 and 50 kDa [32]. Further hydrolysis resulted in low Mw corn arabinoxylan at 2.5 kDa, within the expected range (2–10 kDa) for extensively degraded arabinoxylan [20]. The Mw values reported in this study correspond to estimated Mw obtained from HPSEC-ELSD with pullulan as standards, a very useful methodology applied for the estimation of Mw of polysaccharides of different nature [47]. It should be noted that this approach provides an estimation based on hydrodynamic volume and does not account for potential differences in macromolecular conformation. Since pullulan is a linear polysaccharide, calibration with this standard may lead to deviations when applied to arabinoxylans, which can present varying degrees of branching and structural complexity. Therefore, the values obtained reflect an estimation of Mw and do not provide specific information regarding the branching pattern or molecular architecture of arabinoxylans. Among the other fiber samples, resistant maltodextrin (1.9 kDa) and inulin (0.75 kDa) exhibited the lowest data. These values are also in agreement with published data, where resistant maltodextrins typically range from 1 to 5 kDa [57, 78], and short-chain inulin falls between 0.5 and 2 kDa [37, 60]. These variations in Mw across the fiber types, reflecting different degrees of polymerization and processing, could be critical for understanding their respective physicochemical properties and functional effects.

### Carbohydrate composition of fiber samples

Table 2 presents the soluble fraction (before acid hydrolysis) and the monomeric composition (after acid hydrolysis) of various fiber samples, highlighting the structural differences between fibers and how they break down under acidic conditions.

Corn arabinoxylans exhibit varying carbohydrate compositions depending on their Mw. Before hydrolysis, high and medium-Mw arabinoxylans contained no detectable monosaccharides or oligosaccharides, indicating highly complex structures resistant to spontaneous breakdown. However, low Mw arabinoxylan showed significant levels of xylose (76.57%) and arabinose (23.43%), suggesting increased accessibility of its sugar components as the Mw decreases. After acid hydrolysis, the monomeric composition of high Mw arabinoxylan was predominantly made up of arabinose (76.29%) and xylose (9.59%), along with small amounts of galactose (6.69%), galacturonic acid (4.37%), and mannose (1.03%). The presence of these additional sugars could suggest that the high Mw variant retains a complex structure with some degree of polymerization even after acid hydrolysis with TFA. In contrast, medium Mw arabinoxylan showed a more balanced distribution of xylose (43.82%) and arabinose (32.16%), along with glucose (5.18%) and mannose (11.48%). The release of these additional sugars could imply a less complex structure compared to the high Mw form, allowing for greater efficiency in hydrolysis. Low Mw arabinoxylan exhibited a higher proportion of arabinose (58.57%) as compared to xylose (35.18%), confirming its increased susceptibility to acid hydrolysis. Small amounts of glucose (1.71%) and mannose (0.13%) were also detected, further supporting its simpler, more accessible carbohydrate structure. The variation in sugar composition across Mw highlights the structural differences within arabinoxylans [59]. Higher Mw fractions maintain a more intricate polysaccharide arrangement, possibly due to greater cross-linking with ferulic acid or interactions with other cell wall components [52]. The observed shift in sugar composition after hydrolysis could indicate that arabinoxylans undergo partial degradation, releasing their predominant pentose sugars (arabinose and xylose) [19, 63], while minor components such as galactose and galacturonic acid that could be linked to side chains or residual pectic substances [42, 75].

The carbohydrate soluble fraction detected before hydrolysis of resistant maltodextrin was composed primarily of glucose (18.66%) and trisaccharides (78.12%), with minor levels of maltose (3.23%). This suggests that the fiber retains some oligosaccharide structures derived from starch hydrolysis. Following acid hydrolysis with TFA, resistant maltodextrin was almost entirely converted to glucose (99.25%), with a minimal presence of fructose (0.75%). This likely suggests that its structure, as a highly hydrolyzable starch derivative, readily breaks down into glucose monomers under acidic conditions [3]. The significant shift in composition post-hydrolysis could indicate the susceptibility of resistant maltodextrin to acid hydrolysis, which effectively could depolymerize its oligosaccharide chains. This observation agrees with previous studies showing that resistant maltodextrins, while resistant to enzymatic digestion in the small intestine, can be readily hydrolyzed under acidic conditions, resulting in free glucose molecules [64].

As a fructan-type fiber, inulin’s pre-hydrolysis composition was dominated by fructose (35.90%) and sucrose (55.29%), with small amounts of glucose (7.91%) and mannose (0.90%). The presence of sucrose highlights inulin’s structure, which consists mainly of fructose chains terminating with glucose or sucrose units. The monomeric composition observed after hydrolysis of inulin was a carbohydrate profile mainly consisting of glucose (83.31%) and fructose (16.69%). Interestingly, the high glucose content suggests potential conversion of fructose into glucose during acidic conditions, possibly due to enolization or β-elimination reactions [14]. This phenomenon has been observed in other fructan hydrolysis studies [10]. Inulin’s transformation during hydrolysis suggests structural fragility under acidic conditions, where its β-(2→1) glycosidic linkages break down rapidly [76]. The unexpected predominance of glucose post-hydrolysis could be ascribed to the partial rearrangement of fructose molecules [67]. The presence of sucrose in the pre-hydrolysis composition could indicate that inulin samples contain both free disaccharides and polymerized fructose chains, further supporting its role as a prebiotic fiber with a complex structural makeup [5].

**Table 2:** Carbohydrate composition (%) of various fibers before (soluble fraction) and after acid hydrolysis (monomeric composition)

Sample	High molecular weight corn arabinoxylan		Medium molecular weight corn arabinoxylan		Low molecular weight corn arabinoxylan		Resistant maltodextrin		Inulin	
	Before	After	Before	After	Before	After	Before	After	Before	After
Xylose	–	9.59 ± 0.16	–	43.82 ± 0.80	76.57 ± 1.30	35.18 ± 3.04	–	–	–	–
Arabinose	–	76.29 ± 0.54	–	32.16 ± 0.71	23.43 ± 1.30	58.57 ± 2.95	–	–	–	–
Glucose	–	0.02 ± 0.00	–	5.18 ± 0.05	–	1.71 ± 0.03	18.66 ± 1.21	99.25 ± 0.02	7.91 ± 0.02	83.31 ± 1.74
Fructose	–	0.10 ± 0.00	–	5.79 ± 0.03	–	0.33 ± 0.01	–	0.75 ± 0.02	35.90 ± 0.15	16.69 ± 1.74
Mannose	–	1.03 ± 0.11	–	11.48 ± 0.00	–	0.13 ± 0.02	–	–	0.90 ± 0.01	–
Maltose	–	–	–	–	–	–	3.23 ± 0.11	–	–	–
Trisaccharide	–	–	–	–	–	–	78.12 ± 1.33	–	–	–
Sucrose	–	–	-	–	–	–	–	–	55.29 ± 0.12	–
Rhamnose	–	1.92 ± 0.56	-	0.35 ± 0.02	–	0.18 ± 0.04	–	–	–	–
Galactose	–	6.69 ± 0.29	-	0.12 ± 0.00	–	2.84 ± 0.04	–	–	–	–
Galacturonic acid	–	4.37 ± 0.53	-	1.10 ± 0.02	–	1.07 ± 0.04	–	–	–	–

Undetectable (–)

### Impact of digestion on carbohydrate composition

Table 3 presents the carbohydrate composition of various fiber samples before and after a 4-h digestion period by InfoGest + RSIE. The analysis highlights the transformation of carbohydrates during digestion and provides insight into how different fiber types react under simulated digestive conditions.

At the onset of digestion, corn arabinoxylan contained a range of carbohydrates, including xylose, arabinose, glucose, and others. Notably, higher levels of lactose were detected, which can be attributed to its role as a component of enzyme preparation. After a 4-h digestion period, the levels of xylose, arabinose, and rhamnose remained relatively stable, while a significant increase in galactose was observed. This increase is likely due to the hydrolysis of lactose by the lactase activity of pancreatin and RSIE present in the enzyme mixture [24]. The acidic pH of gastric juice and the enzymatic activity of pepsin further could contribute to the breakdown of polysaccharide structures, releasing glucose and other carbohydrates. As a result, glucose levels increased, suggesting the hydrolysis of higher Mw carbohydrates containing glucose. Moreover, trisaccharides were detected, indicating the possible breakdown of polysaccharides with a degree of polymerization greater than 3 [74].

Interestingly, while the Mw of corn arabinoxylan affected the carbohydrate composition during digestion, all Mw variants show a similar trend in carbohydrate transformation. The low Mw corn arabinoxylan, however, showed higher quantities of carbohydrates such as xylose, arabinose, and mannose before digestion. After 4 h, glucose and arabinose decreased, while maltose and trisaccharides increased. Xylose content also declined, and fructose and mannose were very low and did not show a significant change during digestion. For medium Mw corn arabinoxylan, glucose, arabinose, and traces of rhamnose were present before digestion, but other carbohydrates were minimal. After digestion, the amounts of glucose and trisaccharides increased significantly, while arabinose and rhamnose decreased. The low Mw corn arabinoxylan had a similar trend, with glucose, arabinose, xylose, and small amounts of mannose before digestion. After 4 h, glucose and arabinose levels dropped, while trisaccharides and glucose levels rose. Xylose decreased, and mannose remained stable.

Resistant maltodextrin also went through significant changes after 4 h of digestion. As anticipated, the glucose content increased probably due to the action of α-amylases in both pancreatin and RSIE. Like the corn arabinoxylan samples, higher degrees of polymerization carbohydrates underwent hydrolysis, increasing lower degrees of polymerization carbohydrates like trisaccharides, maltose, and glucose. Lactose from the enzyme preparations was also hydrolyzed, releasing glucose and galactose [12, 24]. Additionally, a small amount of fructose that increased slightly by the end of the 4-h digestion period was detected in the resistant maltodextrin samples. This result was likely due to impurities in the sample.

For inulin, at time 0, fructose was the dominant carbohydrate, followed by glucose, with smaller amounts of arabinose and sucrose. After 4 h of digestion, fructose content decreased substantially, while glucose levels rose. Arabinose and sucrose also decreased during digestion, likely due to the breakdown of inulin’s fructose-rich structure. The partial hydrolysis of sucrose, aided by the acidic environment of gastric juice and the enzymatic activity of RSIE, likely contributed to these changes. These findings are in line with previous studies showing that inulin-type fructans and fructooligosaccharides exhibit high resistance to enzymatic digestion. The strong fructosyl-fructose bonds characteristic of inulin limits its breakdown by intestinal enzymes, supporting its classification as a prebiotic carbohydrate. However, as demonstrated in Gallego-Lobillo et al. [23] even recognized non-digestible carbohydrates can undergo partial hydrolysis when exposed to small intestinal enzyme preparations. This suggests that although inulin remains largely intact, some degree of degradation occurs, potentially affecting its fermentability and physiological impact [23]. Like the other fiber types, an increase in galactose was observed, likely due to the lactase activity that hydrolyzes lactose in enzyme preparation. The mannose remained largely unaffected throughout digestion.

**Table 3:** Carbohydrates composition (mg/g of sample) before and after digestion under simulated physiological conditions using infogest and RSIE

	High molecular weight corn arabinoxylan			Medium molecular weight corn arabinoxylan			Low molecular weight corn arabinoxylan			Resistant maltodextrin			Inulin		
Time (h)	0	4	Sig.	0	4	Sig.	0	4	Sig.	0	4	Sig.	0	4	Sig.
Fructose	–	–	–	–	–	-	0.36 ± 0.05	0.52 ± 0.06	ns	0.24 ± 0.01	0.81 ± 0.31	ns	39.24 ± 2.77	43.37 ± 0.22	ns
Galactose	0.43 ± 0.01	3.38 ± 0.10	***	0.0 ± 0.0	3.11 ± 0.19	**	0.12 ± 0.01	3.09 ± 0.05	***	0.0 ± 0.0	3.79 ± 0.52	***	0.15 ± 0.02	3.28 ± 0.03	***
Glucose	0.54 ± 0.02	7.68 ± 0.11	***	0.58 ± 0.01	18.26 ± 0.98	**	0.73 ± 0.04	6.05 ± 0.07	***	4.95 ± 0.08	31.70 ± 0.06	***	4.31 ± 0.12	12.77 ± 0.0	***
Lactose	24.09 ± 0.13	17.38 ± 0.75	***	19.66 ± 0.24	17.75 ± 0.46	**	20.91 ± 1.88	20.56 ± 1.09	ns	20.20 ± 0.48	19.38 ± 0.95	ns	39.94 ± 2.51	27.99 ± 0.85	*
Maltose	–	–	–	–	–	–	–	–	–	1.32 ± 0.04	0.64 ± 0.14	**	–	–	–
Trisaccharide	0.0 ± 0.0	21.23 ± 0.18	***	0.0 ± 0.0	23.35 ± 0.76	**	0.0 ± 0.0	20.63 ± 1.15	***	0.0 ± 0.0	27.69 ± 2.67	*	–	–	–
Xylose	1.16 ± 0.02	0.81 ± 0.65	ns	0.0 ± 0.0	0.55 ± 0.06	**	6.50 ± 0.69	3.61 ± 0.12	*	–	–	–	–	–	–
Arabinose	5.65 ± 0.06	3.12 ± 0.63	ns	0.47 ± 0.08	1.28 ± 0.05	**	5.99 ± 0.20	3.16 ± 0.05	**	–	–	–	–	–	–
Rhamnose	1.43 ± 0.00	0.78 ± 0.07	***	–	–	–	0.37 ± 0.05	0.52 ± 0.06	*	–	–	–	–	–	–
Mannose	–	–	–	–	–	–	0.13 ± 0.01	0.17 ± 0.01	ns	–	–	–	0.28 ± 0.01	0.46 ± 0.01	**
Sucrose	–	–	–	–	–	–	–	–		–	–	–	26.91 ± 1.60	7.81 ± 1.27	***

(–) undetectable, *: *p* ≤ 0.05, **: *p* ≤ 0.01, ***: *p* ≤ 0.001, ns: non-significant

### Rate of simple sugars release

The rate of simple sugar release following digestion varied significantly across the polysaccharides tested, as summarized in Table 4. These differences highlight the role of carbohydrate structure in influencing the speed and extent of sugar release during digestion.

The high Mw corn arabinoxylan showed an increase in simple sugar release, rising from 0.96 mg/g at 0 h to 11.06 mg/g at 4 h, corresponding to the lowest release rate (2.53 mg/g/h). This is likely due to the complex molecular structure of high Mw arabinoxylan as mentioned in Sect. 3.2. (arabinose/xylose ratio, degree of branching, ferulate content), which resists enzymatic breakdown [54, 58]. Such resistance to hydrolysis could result in a more gradual sugar release, which may be beneficial for individuals seeking steady sugar availability, such as those managing blood sugar levels or those with diabetes [26]. The slower digestion and absorption of this polysaccharide may help prevent rapid sugar spikes and contribute to more stable energy levels [30].

In contrast, the medium Mw arabinoxylan exhibited a much faster sugar release, increasing from 0.58 mg/g to 21.37 mg/g, with a release rate of 5.20 mg/g/h. This suggests that medium Mw arabinoxylan could be more easily hydrolyzed by digestive enzymes, resulting in a quicker release of simple sugars. A key structural difference contributing to this faster release is the significantly lower arabinose-to-xylose ratio of 0.73 in medium Mw arabinoxylan, as compared to 8 in high Mw arabinoxylan and 1.66 in low Mw arabinoxylan (based on Table 2). Arabinose side chains create steric hindrance, limiting enzyme access to the xylan backbone [46]. The lower presence of arabinose in medium Mw arabinoxylan likely reduces this steric hindrance, allowing for more efficient enzymatic hydrolysis of the xylan backbone and a faster release of sugars [41]. This structural characteristic could make medium Mw arabinoxylan a potential candidate for applications requiring a more rapid energy source.

Similarly to high Mw arabinoxylan, low Mw arabinoxylan also exhibited a slower sugar release after hydrolysis. The sugar content increased from 1.35 mg/g to 9.84 mg/g, resulting in a release rate of 2.12 mg/g/h. While this rate is lower than that of medium Mw arabinoxylan, it still reflects a more controlled digestion process. The slower breakdown and sugar release of low Mw arabinoxylan may be attributed to its higher arabinose-to-xylose ratio of 1.66 compared to medium molecular weight arabinoxylan. Although low Mw arabinoxylan has a lower Mw, its slightly higher arabinose substitution may create mild steric effects, reduce enzyme efficiency and lead to a more gradual sugar release [77]. Additionally, differences in chain conformation and solubility between low Mw arabinoxylan and medium Mw arabinoxylan could further influence enzymatic accessibility, contributing to the observed differences in hydrolysis rates [51].

Resistant maltodextrin demonstrated a significant increase in sugar release, from 5.19 mg/g to 32.50 mg/g, with a release rate of 6.83 mg/g/h. This relatively rapid release suggests that maltodextrin can serve as a quick energy source. However, its branched structure with α-(1→4) and α-(1→6) glycosidic linkages could contribute to its partial resistance to enzymatic hydrolysis, leading to incomplete digestion in the small intestine [12]. As a result, a portion of resistant maltodextrin could reach the colon, where it undergoes fermentation by gut microbiota, producing short-chain fatty acids rather than contributing directly to blood glucose levels [56].

In contrast, inulin exhibited a more gradual sugar release, increasing from 43.98 mg/g to 59.88 mg/g, with a release rate of 3.97 mg/g per hour. This controlled hydrolysis can be attributed to inulin’s β-(2→1) fructan structure, which resists enzymatic breakdown in the small intestine. Instead, inulin undergoes limited hydrolysis by β-fructosidases, while its longer-chain fractions remain intact and are selectively fermented in the colon [66]. The fermentation process could generate prebiotic effects, supporting beneficial gut bacteria while minimizing rapid fluctuations in blood glucose levels [15].

These differences can be interpreted through a mechanistic framework: polysaccharides reduce diffusion and mass transfer, impede mixing, block enzyme active sites, induce conformational changes, and form aggregates or surface bonds that immobilize substrates. The extent of inhibition depends on polysaccharide concentration, viscosity, molecular structure, and substrate properties, including molecular weight and conformation [33]. Fibers with higher molecular weight and specific branching patterns, such as high Mw arabinoxylans, resistant maltodextrin, and inulin, showed slower sugar release, whereas medium Mw arabinoxylans with lower steric hindrance were digested more rapidly.

In summary, no significant differences were observed between high and low Mw corn arabinoxylans, both releasing sugars more slowly than inulin. Medium Mw arabinoxylan exhibited release rates closer to resistant maltodextrin, highlighting the combined influence of molecular weight, branching, and arabinose substitution on functional digestibility.

**Table 4:** Simple sugars (mg/g of sample) for each fiber before (0 h) and after (4 h) digestion

	Time (h)	Significance	Sum of simple sugars (mg/g)	Rate of simple sugar release (mg/g/h)
High molecular weight corn arabinoxylan	0	***	0.96 ± 0.00	2.53 ± 0.54^a^
	4		11.06 ± 0.22	
Medium molecular weight corn arabinoxylan	0	***	0.58 ± 0.01	5.20 ± 0.19^c^
	4		21.37 ± 0.78	
Low molecular weight corn arabinoxylan	0	***	1.35 ± 0.09	2.12 ± 0.04^a^
	4		9.84 ± 0.06	
Resistant maltodextrin	0	***	5.19 ± 0.09	6.83 ± 0.07^d^
	4		32.50 ± 0.37	
Inulin	0	*	43.98 ± 2.91	3.97 ± 0.68^b^
	4		59.88 ± 0.17	

*: *p* ≤ 0.05, **: *p* ≤ 0.01, ***: *p* ≤ 0.001; different superscript letters are significantly different (*p* ≤ 0.05)

## Conclusion

In conclusion, our study suggests that corn arabinoxylans exhibit structural diversity that strongly influences their digestive behavior. Carbohydrate composition analysis revealed distinct polymerization patterns across high, medium, and low molecular weight corn arabinoxylans, which correlate with their resistance to enzymatic breakdown. High-Mw arabinoxylans remained largely intact during digestion, releasing sugar slowly. Medium-Mw fibers were hydrolyzed more readily, releasing sugars faster, while low-Mw fibers showed intermediate sugar release. These findings afford information on plausible relationship between molecular weight, structural branching, and digestibility, emphasizing that fiber structure can govern enzymatic resistance and subsequent sugar release. While health-related outcomes such as blood sugar modulation are suggested by these digestive patterns, they should be interpreted cautiously, as this study used in vitro models rather than in vivo trials. Limitations include the use of static digestion models and the focus on a limited number of fiber types, which may not capture all physiological complexities. Overall, the results highlight the potential for selecting corn arabinoxylans with specific molecular characteristics to tailor functional food and pharmaceutical applications (e.g. glycemic control, improvement of gastrointestinal function) optimize digestion profiles and enhance mechanistic understanding of fiber structure–function relationships.

## Supplementary Information

Below is the link to the electronic supplementary material.

Supplementary Material 1

Supplementary Material 2

Supplementary Material 3

Supplementary Material 4

Supplementary Material 5

Supplementary Material 6

## Data Availability

The datasets generated during the current study are available from the corresponding author on reasonable request.
