# Supplementary material for: Impact of fiber molecular structure on resistance to digestion using the infogest and rat small intestine extract protocols
Source: Eur J Nutr. 2025 Dec 6;65(1):3. doi: 10.1007/s00394-025-03853-0 (PMC12681484; doi:10.1007/s00394-025-03853-0)
Supplement: Supplementary file 1 — Supplementary Material 1 [file 394_2025_3853_MOESM1_ESM.docx]

**Table S1:** Enzymatic activities and protein content of commercial pancreatin and rat small intestinal extract (RSIE) preparations

|  | **Pancreatin** | **RSIE** | **Unit** |
| --- | --- | --- | --- |
| Protein Content | 33.33 | 47.13 | mg |
| Lactase Activity | 0.1 | 18.01 | U |
| Sucrase Activity | - | 57.80 | U |
| Inulinase Activity | 0.42 | 6.35 | U |
| Maltase Activity | 36.13 | 250.34 | U |
| Alpha-Amylase Activity | 10.76 | 18.17 | U |
| Trypsin Activity (InfoGest Protocol) | 2.64 | - | U/mL |
