# Supplementary material for: Impact of fiber molecular structure on resistance to digestion using the infogest and rat small intestine extract protocols
Source: Eur J Nutr. 2025 Dec 6;65(1):3. doi: 10.1007/s00394-025-03853-0 (PMC12681484; doi:10.1007/s00394-025-03853-0)
Supplement: Supplementary file 2 — Supplementary Material 2 [file 394_2025_3853_MOESM2_ESM.docx]

**Table S2**: Carbohydrate content of commercial pancreatin and rat small intestinal extract (RSIE) preparations (mg/g sample)

| **Preparation** | **Time** | **Galactose** | **Glucose** | **Lactose** | **Total Carbohydrates** |
| --- | --- | --- | --- | --- | --- |
| Pancreatin | T0 | 0.65 | 0.53 | 225.33 | 226.51 |
| Pancreatin | Tf | 0.67 | 0.52 | 221.41 | 222.60 |
| RSIE | T0 | – | 0.44 | 0.71 | 1.15 |
| RSIE | Tf | – | 0.58 | 0.62 | 1.09 |

T0 = initial timepoint; Tf = final timepoint
