# Supplementary figures and images for: Impact of fiber molecular structure on resistance to digestion using the infogest and rat small intestine extract protocols
Source: Eur J Nutr. 2025 Dec 6;65(1):3. doi: 10.1007/s00394-025-03853-0 (PMC12681484; doi:10.1007/s00394-025-03853-0)

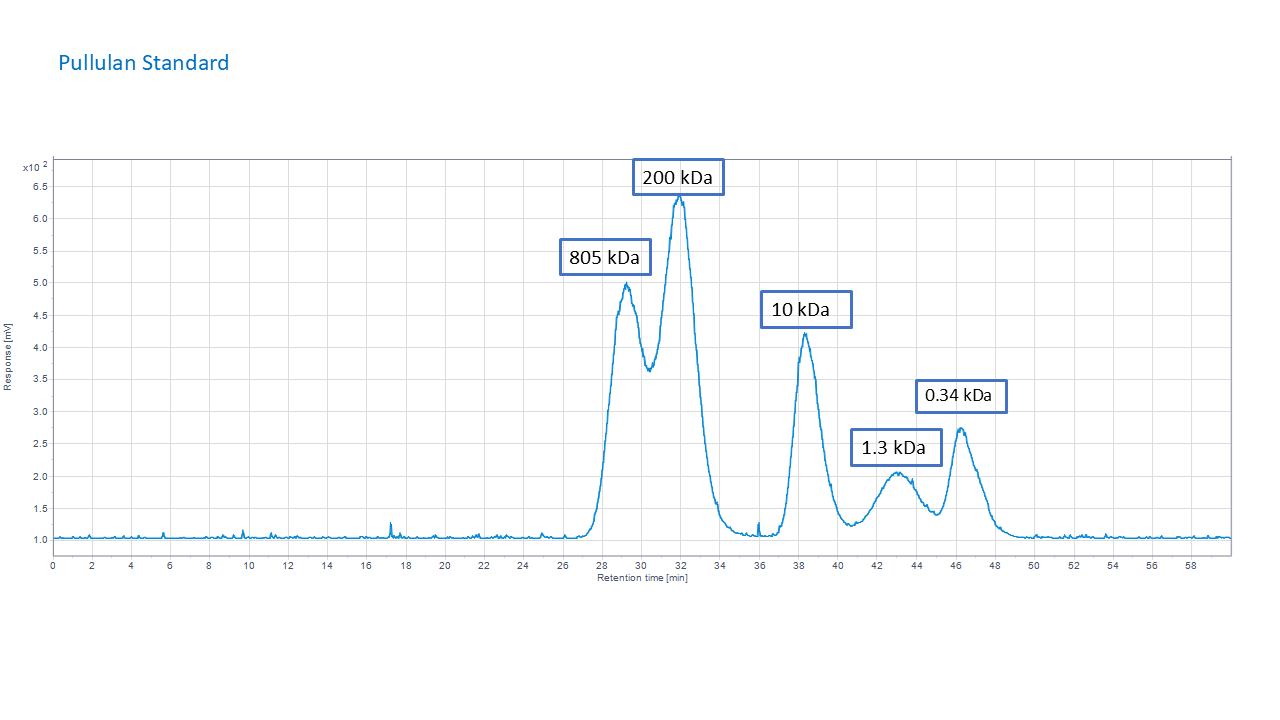

Supplement: Supplementary file 3 — Supplementary Material 3 [file 394_2025_3853_MOESM3_ESM.tif]

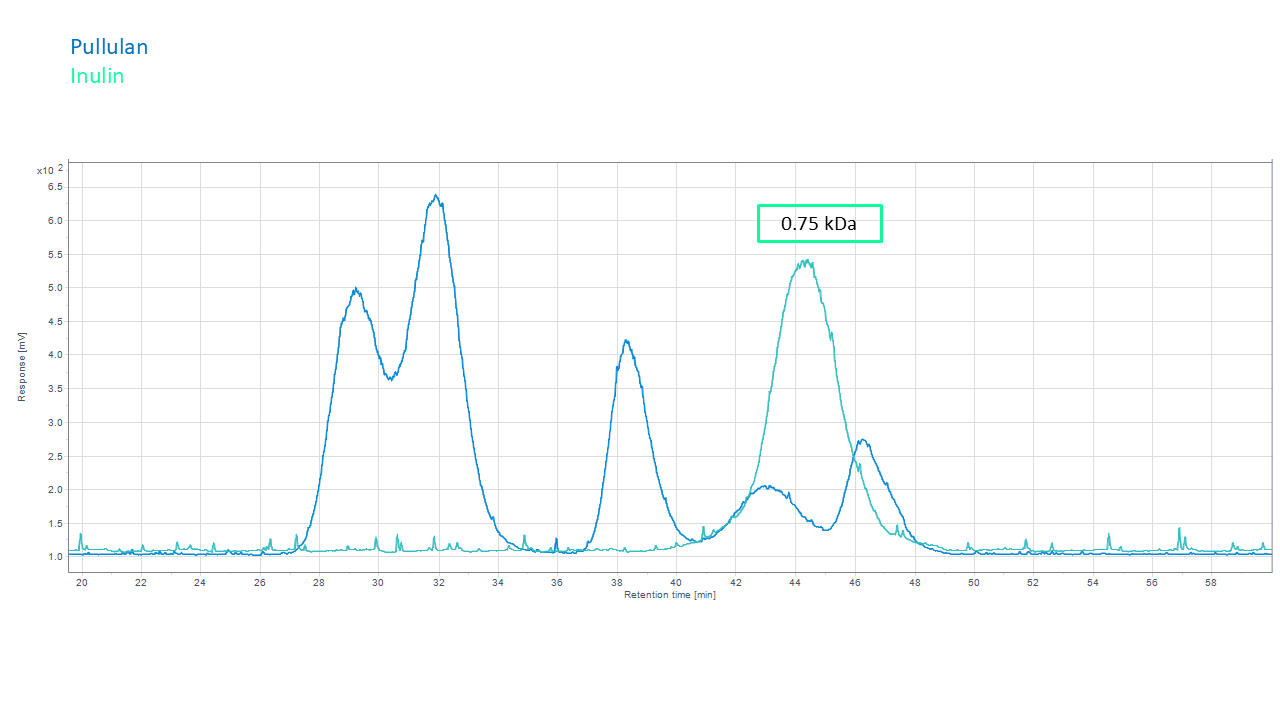

Supplement: Supplementary file 4 — Supplementary Material 4 [file 394_2025_3853_MOESM4_ESM.tif]

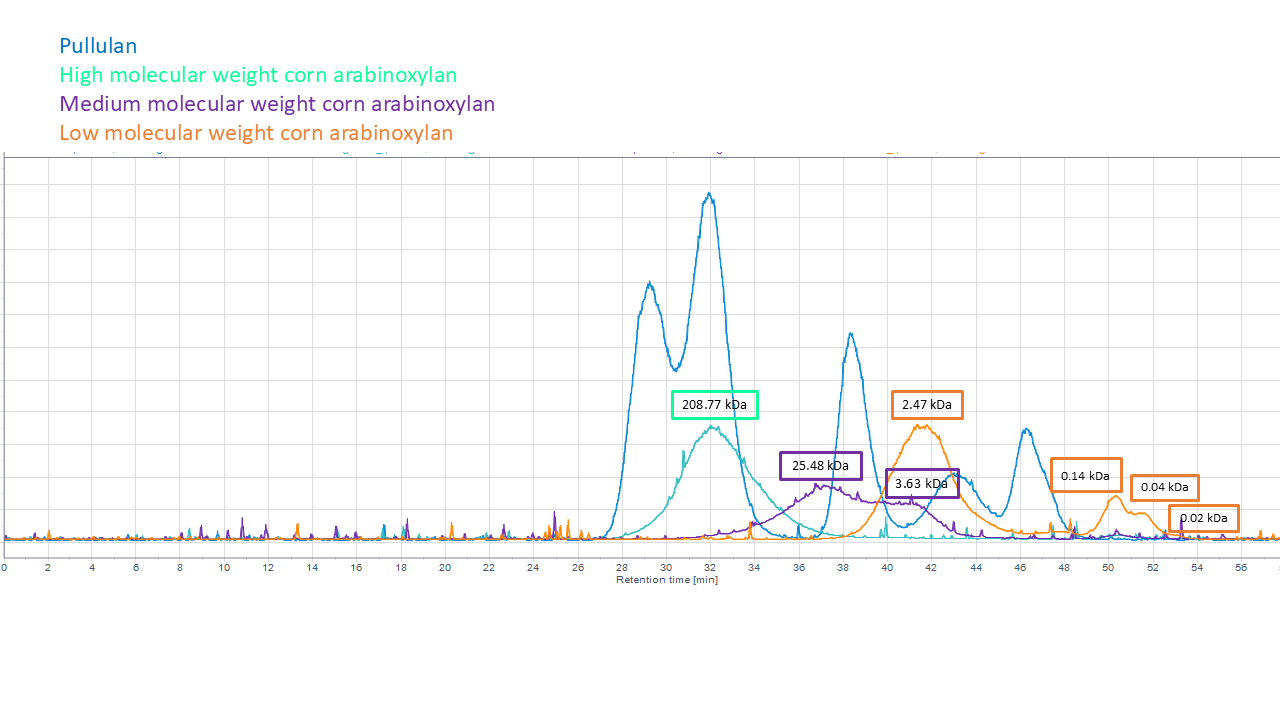

Supplement: Supplementary file 5 — Supplementary Material 5 [file 394_2025_3853_MOESM5_ESM.tif]

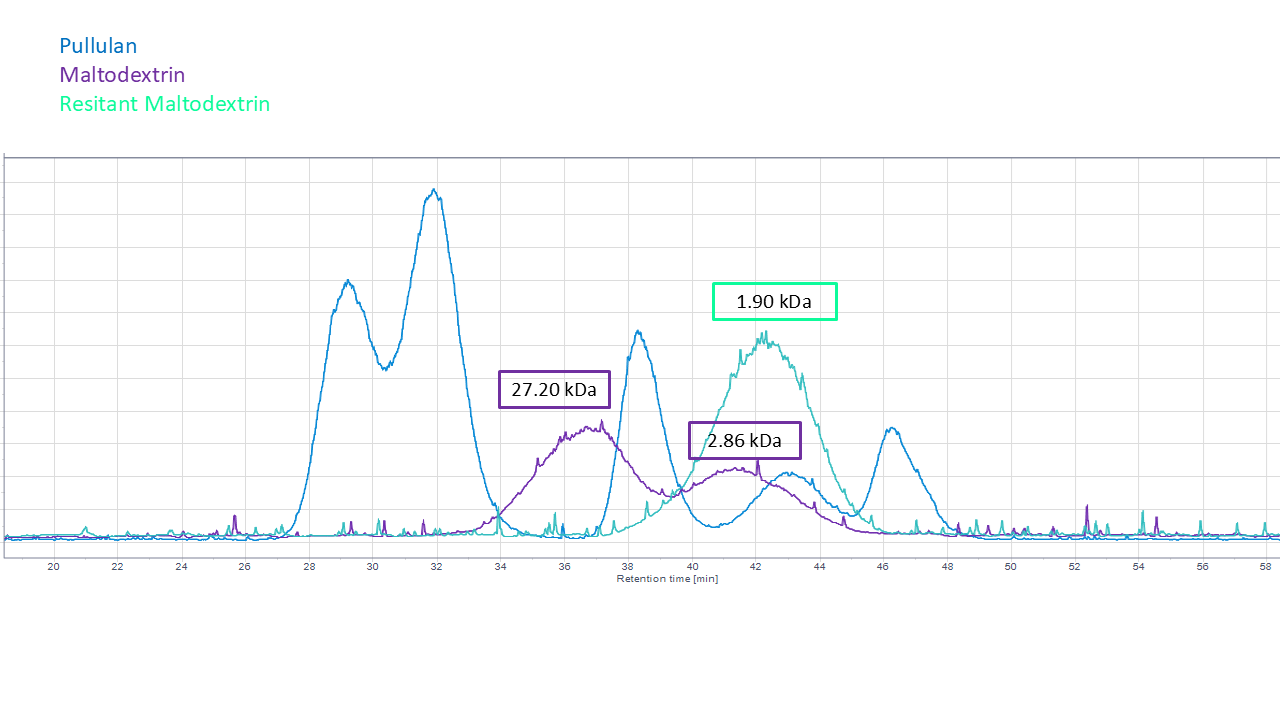

Supplement: Supplementary file 6 — Supplementary Material 6 [file 394_2025_3853_MOESM6_ESM.tif]
